# Macrophage type 2 differentiation in a patient with laryngeal squamous cell carcinoma and metastatic prostate adenocarcinoma to the cervical lymph nodes

**DOI:** 10.1186/s40425-017-0264-z

**Published:** 2017-07-18

**Authors:** Michael C. Topf, Madalina Tuluc, Larry A. Harshyne, Adam Luginbuhl

**Affiliations:** 1 Department of Otolaryngology—Head and Neck Surgery, Thomas Jefferson University, 925 Chestnut Street, 6th Floor, Philadelphia, PA 19107 USA; 2 Department of Pathology, Anatomy, and Cell Biology, Thomas Jefferson University, 285 Main Building, 132 S. 10th Street, Philadelphia, PA 19107 USA; 3 Department of Neurosurgery, Thomas Jefferson University, 1020 Locust Street Suite 454, Philadelphia, PA 19107 USA

**Keywords:** M2 macrophages, CD163, Tumor microenvironment

## Abstract

**Background:**

The tumor microenvironment often polarizes infiltrating macrophages towards a type 2, or M2 phenotype, that is characterized by expression of various cysteine-rich, scavenger receptors, including CD163. The primary function of M2 macrophages is to facilitate wound healing. As such, they are capable of providing metabolic support to a growing tumor, neovascularization, as well as protection from cytotoxic T cells. The tumor microenvironment contains a milieu of secreted factors and vesicles, which in certain circumstances can gain access to lymphatic vessels that drain to local lymph nodes.

**Case presentation:**

We report a 59-year-old male with recurrent T4 squamous cell carcinoma (SCC) of the larynx with synchronous prostate adenocarcinoma confined to the prostate and regional pelvic lymph nodes, without metastatic disease. The patient underwent salvage total laryngectomy and bilateral neck dissection with final pathology revealing a recurrent moderately differentiated SCC involving the larynx as well as prostate cancer in draining level 4 cervical lymph nodes bilaterally. CD163 staining was performed on the primary tumor, a negative draining lymph node, and a level four lymph node with a focus of metastatic prostate cancer and compared to benign controls. The negative draining lymph node demonstrated a large CD163 population of cells as did the interface of the focus of prostate cancer and surrounding lymph node. CD163 levels were markedly increased in this patient compared to benign lymph node controls. The macrophage differentiation at the primary tumor in the larynx was strongly CD163 positive supporting an immune permissive environment for tumor growth and metastasis.

**Conclusion:**

We describe a unique case of solitary metastatic prostate cancer to cervical lymph nodes in the setting of a laryngeal cancer. These observations suggest that SCC-derived factors drive a tumor-supportive environment in draining lymph nodes dominated by an overwhelming number of CD163+, M2 macrophages. Lymph nodes that are ‘primed’ by SCC differentiation to M2 phenotype may be at higher risk of harboring metastases.

## Background

Macrophages are innate immune cells that are influenced by factors present in the local microenvironment to guide proper immune programs. In this capacity, macrophages are found to have two main phenotypes: macrophage 1 (M1) and macrophage 2 (M2), which correspond to respective T-helper cell subsets [1–4]. M1 macrophages are activated by IFN-γ and secrete pro-inflammatory cytokines such as IL-12 and tumor-necrosis factor alpha, which support anti-tumor immune responses. Conversely, M2 macrophages are activated by IL-4, IL-10, and IL-13 and secrete anti-inflammatory cytokines such as TGFβ and IL10, and angiogenic factors such angiopoietin and VEGF, which promote tumor growth, invasion, and metastases. The balance between M1 and M2 macrophages is one of the many factors that allow a transition from a cytotoxic lethal immune environment of a normal lymph node to an immunotolerant environment that allows tumors metastasis [1, 4].

Tumor-associated macrophages (TAMs) are key mediators in the tumor microenvironment by modulating metabolic factor production and cytokine profiles within the tumor microenvironment [1–4]. TAMs possess a predominant M2 phenotype indicative of local immune suppression [2, 5]. Previous studies have investigated the balance between M1 and M2 macrophages in head and neck squamous cell carcinoma (HNSCC) and found that the majority of TAMs are predominantly the M2 phenotype [1, 6, 7]. Furthermore, increased M2 polarization has been associated with decreased survival in HNSCC [8–11]. The CD163 protein belongs to a scavenger receptor cysteine-rich family that is expressed on the surface of M2 macrophages and can be used as an immunohistochemical marker for these cells [12].

In this case report, we describe a patient with a unique clinical presentation of metastatic prostate cancer to bilateral cervical lymph nodes in the setting of laryngeal squamous cell carcinoma (SCC). We further investigated the M2 differentiation of the draining cervical lymph nodes. We hypothesize that the patient’s laryngeal SCC was able to skew the local immune microenvironment to a permissive atmosphere favoring tumor escape and metastasis, allowing for his prostate cancer to spread to the cervical lymph nodes.

## Case presentation

A 59-year-old male with recurrent T4 N0 SCC of the larynx with synchronous prostate adenocarcinoma was referred to our clinic. His prostate adenocarcinoma was confined to the prostate and regional pelvic lymph nodes, without any known distant metastatic disease, and was being treated with hormone injections. Physical examination showed no evidence of neck disease and pre-operative computed tomography (CT) imaging of the head and neck showed no other evidence of disease. The patient underwent salvage total laryngectomy and bilateral neck dissection with final pathology revealing a recurrent moderately differentiated SCC involving the anterior larynx as well as metastatic prostate cancer in draining level 4 cervical lymph nodes bilaterally. The patient’s positron emission tomography (PET)/CT scan 3 months following salvage laryngectomy was positive for only his known regional pelvic metastatic lymph nodes with no evidence of FDG-avid locoregional tumor recurrence in the head and neck. We did not find circulating adenocarcinoma tumor cells post-operatively on serum analysis (Cell Tracks Analysis). The patient was seen in follow-up 6 months following salvage laryngectomy with no evidence of disease.

## Methods

Two surgical pathologists independently confirmed the diagnosis of metastatic prostate cancer to the cervical lymph nodes. Sections 4 μm thick from formalin fixed paraffin embedded tissue were placed on positively charged slides, dried overnight at 65 °C and then stained with the primary antibody CD163 (MRQ-26 clone, Cell Marque, Rocklin, CA) on an automated platform (Ventana, Tucson AZ). A pathologist reviewed all H&E stained slides as well as positive and negative controls for quality. Slides were uploaded into Aperio Imagescope (Leica Biosystems, Buffalo Grove IL) and annotated by a head and neck pathologist to include only the lymph node portion for analysis. Quantitative analysis of macrophage membrane staining was performed using digital immunohistochemistry (IHC) analysis with Aperio Imagescope to determine the percent of total cells that stained strongly (2+, 3+) for CD163 for each slide. This analytic algorithm has been FDA approved for detecting hormonal receptors in breast cancer. CD163 staining was performed on a benign draining level three lymph node, a level four lymph node with a focus of metastatic prostate cancer and the primary laryngeal tumor. We compared this to 12 benign lymph nodes of patients who underwent parotidectomy for benign pathology.

## Results

**Fig. 1:**
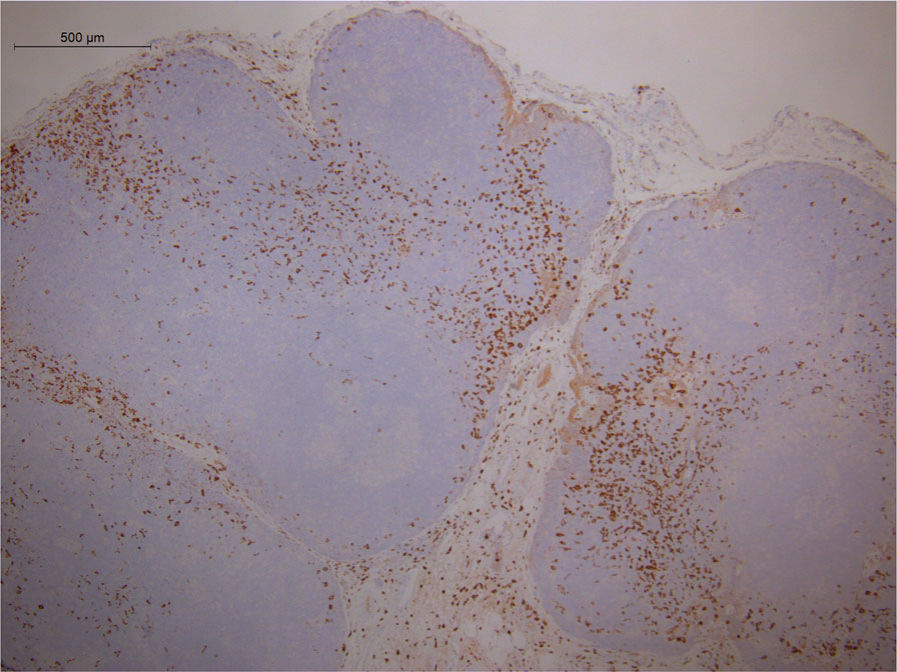
CD163 staining in a reactive lymph node. Immunohistochemical detection of CD163 cells in a benign lymph node of a cancer-free parotidectomy patient demonstrates low levels of M2 differentiation

**Fig. 2:**
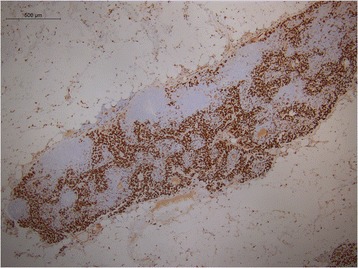
CD163 staining in a negative draining lymph node from the patient with laryngeal SCC and prostate adenocarcinoma metastatic to the cervical lymph nodes. CD163 staining performed on a negative draining lymph node in the SCC patient with metastatic prostate adenocarcinoma to the cervical lymph nodes demonstrates a large CD163 population

**Fig. 3:**
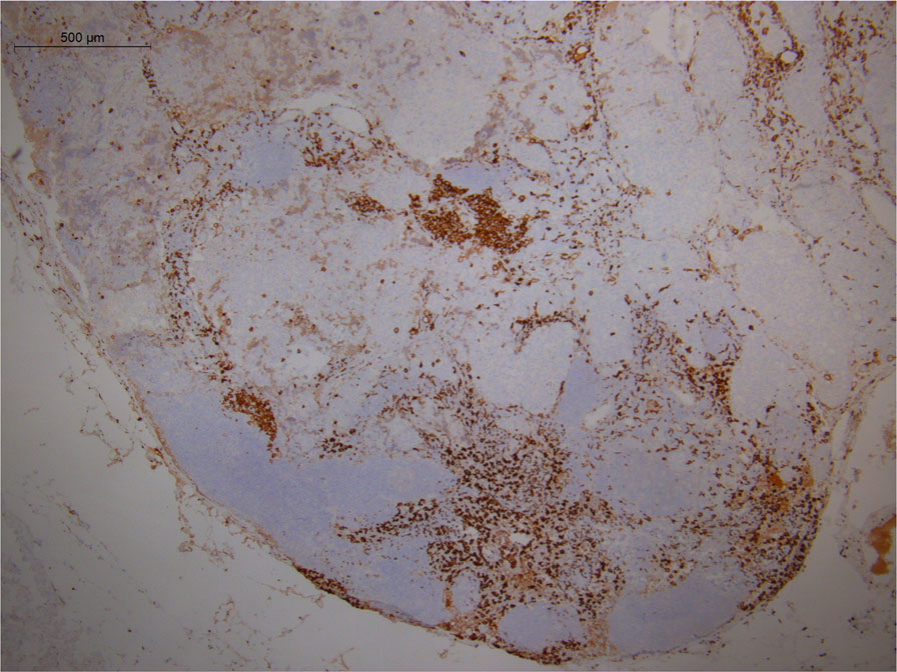
CD163 staining in the lymph node with metastatic prostate adenocarcinoma. CD163 staining performed on the lymph node positive for metastatic prostate adenocarcinoma. The interface of the metastatic focus of prostate adenocarcinoma and the surrounding lymph node demonstrates dense CD163 staining

**Fig. 4:**
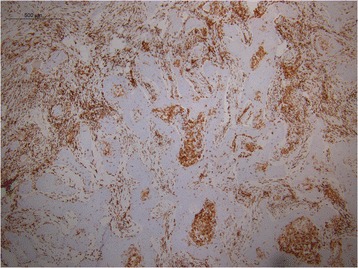
CD163 staining in the primary laryngeal squamous cell carcinoma. CD163 staining performed on the primary SCC tumor in the larynx. Staining was strongly CD163 positive supporting an immune permissive environment for tumor growth and metastasis

Descriptive pattern of staining for CD163 demonstrated a paucity of M2 differentiation in the benign lymph nodes of cancer-free parotidectomy patients (Fig. 1). Conversely, the negative draining lymph node in the SCC patient with metastatic prostate adenocarcinoma to the cervical lymph nodes demonstrated a large CD163 population of cells (Fig. 2), as did the interface of the metastatic focus of prostate cancer and surrounding lymph node (Fig. 3). Additionally, the macrophage differentiation at the primary tumor in the larynx was strongly CD163 positive supporting an immune permissive environment for tumor growth and metastasis (Fig. 4).

Table 1 shows the percent of total cells that stained strongly for CD163 in the 12 benign parotid lymph node controls as well as the CD163 levels of the negative draining lymph node, lymph node with metastatic focus of prostate adenocarcinoma, and primary tumor of our patient. The average CD163 levels of the 12 patients who underwent parotidectomy for benign pathology with benign lymph nodes were 17.56%. In comparison, the percent of cells that stained strongly for CD163 in the negative level three draining lymph node was 62.94%. The CD163 level of the level four lymph node with metastatic focus of prostate carcinoma was 42.91%. The primary tumor had a CD163 level of 50.46%.

**Table 1:** Percent of total cells that stain strongly for CD163

Specimen	CD163 Level
Benign parotid lymph node 1	4.79%
Benign parotid lymph node 2	13.61%
Benign parotid lymph node 3	37.84%
Benign parotid lymph node 4	10.08%
Benign parotid lymph node 5	10.01%
Benign parotid lymph node 6	26.93%
Benign parotid lymph node 7	18.84%
Benign parotid lymph node 8	12.84%
Benign parotid lymph node 9	23.33%
Benign parotid lymph node 10	29.16%
Benign parotid lymph node 11	14.78%
Benign parotid lymph node 12	8.52%
Average benign parotid lymph node	17.56%
Negative draining lymph node	62.94%
Lymph node with metastatic focus of prostate adenocarcinoma	42.91%
Primary tumor	50.46%

## Discussion and conclusions

The incidence of cervical lymph node involvement in patients with prostate cancer has been reported as 0.4% or less and these patients almost uniformly present with widespread metastatic disease [13, 14]. Despite the high incidence and prevalence of prostate cancer, to the best of our knowledge, there are less than 50 cases of prostate cancer metastatic to the cervical lymph nodes reported in the literature [14–21]. Furthermore, the majority of the cases involve a left supraclavicular lymph node (Virchow’s node) [14, 20], and not necessarily lymph nodes in levels I-VI, a more common site of metastasis in head and neck cancers.

We describe a unique case of metastatic prostate cancer to cervical lymph nodes in the setting of a laryngeal SCC. Our patient had no other foci of metastatic disease with contained disease in the prostate and pelvic lymph nodes. Additionally, serum from this patient did not reveal any circulating prostate tumor cells (Cell Tracks Analysis) supporting the argument that there may be a causative relationship with lymph node priming from the laryngeal primary opposed to simply a system overload with high levels of circulating tumor cells. A review of the literature revealed only two similar cases of prostate carcinoma metastatic to the cervical lymph nodes in the setting of concurrent HNSCC [21].

In the present report we show increased CD163 levels, a marker for M2 macrophages, in both the negative draining lymph node, interface of the metastatic focus of prostate cancer and surrounding lymph node, and primary laryngeal tumor. When compared to benign lymph nodes of patients who underwent parotidectomy for benign pathology, the patient in this case report’s negative draining lymph node had CD163 levels that were three times greater. In addition, our patient had high CD163 levels at the primary tumor indicative of a predominant M2 phenotype, which suggests an immune permissive microenvironment for tumor growth and metastasis.

Based on work done by Harshyne et al. in glioblastoma [22], tumors communicate with the surrounding tumor microenvironment via cytokines, exosomes, or other soluble mediators. The elevated M2 macrophages both in the positive lymph node as well as the negative draining lymph node could fit this pattern of immune editing. These observations could suggest that SCC may be able to alter the tumor microenvironment both at the primary tumor and draining lymph nodes favoring tumor escape and metastasis. In the present case, we hypothesize that the patient’s laryngeal SCC was able to communicate with the local immune microenvironment to skew its phenotype to a permissive atmosphere allowing for occasional circulating prostate cancer cells to metastasize to the cervical lymph nodes.

## Abbreviations

CT: Computed tomography
HNSCC: Head and neck squamous cell carcinoma
M1: Macrophage 1
M2: Macrophage 2
PET: Positron emission tomography
SCC: Squamous cell carcinoma
TAM: Tumor associated macrophage
